# Targeting the *miR-19b*/PPP2R5E axis enhances temozolomide response in glioblastoma via ROS-induced DNA damage

**DOI:** 10.1038/s41416-026-03474-2

**Published:** 2026-05-27

**Authors:** Elham Kashani, Martin C. Sadowski, Jaison Phour, Ali Hashemi Gheinani, Kristyna Hlavackova, Stefan Haemmig, Ulrich Baumgartner, Nicole Mueller-Wirth, Carmen Trefny, Bushra Sharf Den Abu Fakher, Evelina Parvanova, Coline Nydegger, Theoni Maragkou, Philippe Schucht, Aurel Perren, Pascal Zinn, Markus Lüdi, Thomas Michael Marti, Philippe Krebs, Erik Vassella

**Affiliations:** 1 Institute of Tissue Medicine and Pathology, University of Bern, Bern, Switzerland; 2 Functional Urology Research Group, Department for BioMedical Research (DBMR), University of Bern, Bern, Switzerland; 3 Department of Neurosurgery, University Hospital Bern, Bern, Switzerland; 4 Department of Neurological Surgery, University of Pittsburgh School of Medicine, Pittsburgh, PA USA; 5 Department of Anesthesiology and Pain Medicine, University Hospital Bern, University of Bern, Bern, Switzerland; 6 Division of General Thoracic Surgery, Inselspital, Bern University Hospital, Bern, Switzerland

**Keywords:** CNS cancer, Double-strand DNA breaks

## Abstract

**Background:**

Glioblastoma is the most aggressive primary brain tumor. Although temozolomide induces DNA damage, its efficacy is limited by intrinsic resistance mechanisms, leading to tumor relapse. In this study we aimed to identify microRNA-mediated resistance mechanisms.

**Methods:**

MicroRNA screens were performed to identify temozolomide resistance mechanisms. Temozolomide response was evaluated using clonogenic and spheroid assays in glioblastoma cell lines and patient-derived primary cells. DNA damage response was assessed by γH2AX foci formation. Cell cycle distribution, senescence and ferroptosis were assessed following *miR-19b* attenuation. An orthotopic xenograft and a syngeneic mouse model were used to confirm findings.

**Results:**

Our screen and subsequent phosphoprotein analysis identified *miR-19b* and its target PPP2R5E, a subunit of the phosphatase PP2A, as modulators of temozolomide response. Attenuation of *miR-19b* upregulated PPP2R5E, inducing genotoxic stress, thereby enhancing temozolomide response. Mechanistically, DNA damage correlated with elevated nuclear ROS, promoting senescence and ferroptosis. Consistently, pharmacological activation of PP2A with FTY720 phenocopied the effects of *miR-19b* suppression. Conversely, knockdown of PPP2R5E reversed temozolomide sensitisation in vitro, in vivo, and ex vivo models, confirming the critical role of the *miR-19b*/PPP2R5E axis in modulating drug response.

**Conclusions:**

Therapeutic targeting of the PPP2R5E/PP2A complexes holds promise for enhanced temozolomide efficacy in glioblastoma.

## Introduction

Glioblastoma (GBM), the most common and aggressive primary brain tumor in adults, presents a significant medical challenge. The current standard of care for patients diagnosed with GBM involves a combination therapy regimen, which includes surgical resection, treatment with the DNA alkylating agent temozolomide (TMZ), and radiation therapy. However, these interventions offer limited clinical benefit, and patients rarely survive beyond 2 years [1].

Different DNA repair pathways may contribute to the poor treatment response observed in GBM patients. Promoter methylation of the O^6^-methylguanine-DNA methyltransferase (*MGMT*) gene, encoding a protein responsible for repairing alkylated DNA, is associated with a better TMZ response [2]. If not repaired by MGMT, these adducts mispair with thymine, inducing futile cycles of mismatch repair and DNA double stranded breaks (DSBs) during DNA replication. Downregulation of mismatch repair (MMR) genes represents a key escape mechanisms from these futile MMR cycles [3, 4]. Additionally, enhanced homologous recombination activity may undermine TMZ efficacy [5, 6]. Beyond DNA repair capacity, glioblastoma cells employ additional adaptive strategies to cope with TMZ-induced cytotoxicity, including neuroplasticity through glioma synapse recruitment [7], a tumor-permissive microenvironment [8], and activation of pro-survival signaling pathways [9]. Collectively, these processes contribute to the challenge of treating GBM effectively.

MicroRNAs (miRNAs) are key post-transcriptional regulators that coordinate gene expression networks and drive resistance mechanisms (for review, see [10]). Accordingly, systematic miRNA screens have become proven powerful tools for identifying dysregulated pathways and uncovering chemoresistance in cancer [11].

In this study, we performed an unbiased, systematic miRNA screen in GBM cell lines and identified *miR-19b* as a critical modulator of TMZ response. We showed that *miR-19b* directly targets *PPP2R5E*, a regulatory subunit of the serine/threonine protein phosphatase PP2A, uncovering a novel mechanism in which *miR-19b* inhibition leads to ROS-mediated DNA damage and sensitises GBM cells to temozolomide.

## Methods

### Cell lines and primary cells

Human primary glioblastoma stem cells (GSC3 and GSC4) were collected intraoperatively from patients with IDH wild-type GBM at MD Anderson Cancer Center [12] and cultured in GSC media consisting of DMEM/F12 supplemented with B27 (LifeTechnologies, USA), epidermal growth factor [20 ng/ml] (EGF; Sigma-Aldrich, USA), and basic fibroblast growth factor [20 ng/ml] (bFGF; PeproTech, USA).

The GBM cell lines U87MG (kindly provided by M.E. Hegi, University of Lausanne, Switzerland), LN18, and LN229 (purchased from ATCC) were cultured as described [13]. CT2A murine glioma cells (kindly provided by G. Hutter (University of Basel, Switzerland) were cultured in high-glucose Dulbecco’s Modified Eagle Medium (DMEM), supplemented with 10% fetal bovine serum (FBS), L-glutamine [2 mM], and 1% penicillin-streptomycin (Pen/Strep) (all Sigma-Aldrich).

All cell lines were genotyped and authenticated by Microsynth (Switzerland), and tested negative for mycoplasma contamination. Unless otherwise stated, cells were treated with 100 μM TMZ (SelleckChem, USA). PP2A activity was induced by treating cells with 10 μM FTY720 (Sigma-Aldrich).

### Patient-derived xenograft (PDX) mouse model

Groups ten of 5-week-old athymic female nude mice (Crl:NU(NCr)-Foxn1nu; Charles River, USA) were anaesthetised and orthotopically injected into the right forebrain with 1-2 × 10^5^ GSC4 cells co-transduced with a beetle luciferase reporter and either sh(ctrl), shPPP2R5E, or αmiR-19b constructs [14]. Mice were randomly assigned to a group at the beginning of the experiment. The operator was blinded for the cell type that was injected orthotopically.

All animals received TMZ at a dose of 66 mg/kg via oral gavage daily for five consecutive days, starting two weeks after tumor engraftment, followed by a 3-week rest period. This treatment cycle was repeated twice for a total of three cycles.

Tumor size was longitudinally monitored using Bioluminescence imaging with the SpectrumCT In Vivo Imaging System (IVIS; PerkinElmer, USA). Signal intensities from the brain and spine regions were quantified using Living Image® software (PerkinElmer) [15]. An a priori power analysis was performed using G*Power 3.1 to determine the required sample size. The significance level (α) was set to 0.05 and statistical power (1-β) to 0.95. The estimated sample size was calculated based on expected differences between two groups, using effect sizes estimated from in vitro data.

Mice were euthanized upon the development of neurological symptoms or severe weight loss. Animals that did not develop tumors or died from unrelated causes (e.g. cases of sudden death without previous clinical signs or neurological symptoms specific to glioblastoma models) were excluded.

### Ex vivo analysis using syngeneic CT2A organotypic brain slices

Organotypic brain slices were prepared essentially as described previously [16, 17]. In brief, brains were removed from 6-week-old male C57BL/6 mice and embedded in 4% low-melting agarose (Carl Roth, Germany). Coronal sections (300 μm thick) were generated using a VT1200 vibratome (Leica, Germany) and transferred into DMEM-high glucose supplemented with 20% heat-inactivated horse serum, 25% sHBSS, and 1% GlutaMAX (all Sigma-Aldrich). Slices were then placed onto 0.4 μm PET transwell membrane inserts (Sigma-Aldrich) and cultured in 6-well plates containing cultivation medium composed of DMEM-high glucose, 10% heat-inactivated horse serum, 12.5% sHBSS, 2mM L-glutamine, 1% Pen/Strep, B27, N-2 supplement (ThermoFisher, USA), 20 ng/ml bFGF, and 20 ng/ml EGF. GBM spheroids were placed onto the brain slices 24 h post-sectioning. TMZ [100 μM] or vehicle control was added six days after spheroid placement. Tumor growth was monitored longitudinally using the IVIS following 15 min of incubation with D-Luciferin [150 mg/kg].

For endpoint analysis, slices were fixed in 4% paraformaldehyde, mounted in Mowiol (Carl Roth, Germany), and subjected to live/dead staining and imaging using the Leica Mica microscope, as previously described [18].

### Constructs, transfections, and transductions

Constructs used in this study are detailed in the supplementary Table 1. Luciferase reporter plasmids were constructed using the pmirGLO Dual-Luciferase miRNA Target Expression Vector (Promega, USA) [19].

Lentivirus production and transduction of GBM cell lines were performed as described [19]. Transient transfection of short RNAs was performed as described [13]. Sequences of oligonucleotides and lentiviral constructs used in this study are listed in supplementary Table 1.

### MiRNA lentiviral screen and next generation sequencing (NGS)

A pooled lenti-miRNA library (System Biosciences, USA, Cat. PMIRHPLVHT-1) was transduced into 5 × 10^5^ cells at a multiplicity of infection (MOI) of 0.3–0.4 to ensure single-copy integration.

Three days post-transduction, cells were selected with 1.5 μg/ml puromycin (Sigma-Aldrich) for three days, followed by treatment with escalating doses of TMZ, starting at 25 μM TMZ and increasing up to 100 μM over time.

For NGS analysis, genomic DNA was extracted and lentiviral fragments encompassing the pre-miRNA sequences were PCR-amplified using the High-Fidelity MasterMix (New England Biolabs (NEB), USA) with 400 ng DNA, 0.5 μM primers, and 1 mM MgCl_2_ for 30 cycles. The PCR product was fragmented using the Next Fast DNA Fragmentation & Library Prep Set (NEB). Libraries (100 ng) were sequenced using an Ion Torrent PGM platform (ThermoFisher). Differential expression analysis was performed using the RNA Sequencing Data Analysis Workflow in CLC Genomics Workbench (Qiagen,Germany), and relative coverage was normalised to total reads.

### MRNA analysis

RNA extraction and reverse transcription quantitative real-time PCR (RT-qPCR) were performed as described [19]. Primer sequences used for RT-qPCR are listed in supplementary Table 1.

### Cell based assays

Luciferase reporter assay, cell viability assays using alamarBlue (ThermoFisher) and Resazurin (Sigma-Aldrich), caspase3/7 activity assays (ApoTox-Glo Triplex kit; Promega), and BrdU incorporation assay (Roche,Switzerland) were performed essentially as described [13].

Live/dead analysis of spheroid cultures was performed as described [18].

For the colony formation assay, cells were treated with 15 μM TMZ for two weeks, starting 16 h after seeding.

To assess ferroptosis susceptibility, 2000 cells were seeded into 96-well plates and treated for 48 h with RSL3 or erastin (SelleckChem), or co-treated with ferrostatin-1 (Sigma-Aldrich) or trolox (Millipore, USA). Treatment response was evaluated using the BrdU incorporation and alamarBlue assays.

Senescence was assessed by senescence-associated β-galactosidase (SA-β-gal) staining using the Senescence β-Galactosidase Staining Kit (Cell Signaling, USA). A total of 3 × 10^4^ cells were seeded in 6-well plates and treated with 100 μM TMZ for 48 h, beginning 16 h post-seeding. At least ten randomly selected fields ( ~ 500 cells) were imaged at 20X magnification using a bright-field microscope.

γH2AX foci formation was assessed by seeding 2000 cells in µ-Plate 96-Well Square Glass Bottom plates (ibidi, Germany; 89626). Cells were fixed with 2% paraformaldehyde at room temperature for 10 min, permeabilized with 0.5% Triton X-100 in PBS, and blocked with 10% BSA/PBS for 1 h. Cells were then incubated overnight at 4 °C with anti-γH2AX primary antibody (1:500, Cell Signaling #2577). Images were acquired using an IN Cell Analyzer 2000 (GE Healthcare Life Sciences, USA), and foci quantification was performed using Cell Profiler (v.4.0.7).

ROS fluorescence imaging by confocal microscopy was performed on live cells seeded in µ-plate 96-well square glass bottom plates (ibidi). Cells were stained with CellROX Deep Red [2.5 μM], MitoTracker Orange [100 nM], and Hoechst 33342 [4 μM] (all from ThermoFisher) in 100 μL staining solution for 45 min in a 5% CO₂ incubator. At least 500 cells per well were imaged (4 images per well, 10X magnification), and ROS quantification was performed using CellProfiler (v4.2.6).

### Western blot, immunohistochemistry and phosphokinase arrays

Western blot procedures and antibodies are described elsewhere [19]. Additional antibodies used in this study include anti-phospho-Histone H2AX (Ser139) (Cell Signaling #2577S, 1:1000) and anti-PPP2R5E [EPR17147]-c-terminal (Abcam, ab198500, 1:1000). For the latter, only 10 µgr total protein was loaded, as lower input was required to maintain signal linearity. Protein levels were normalised to total protein.

Immunohistochemistry (IHC) was performed on formalin-fixed paraffin embedded tissue using the same anti-PPP2R5E antibody clone at a 1:750 dilution and expression analysis was performed using QuPath essentially as described [20]. Tumor annotations and pathology review of the mice and human tissue were assessed in a blinded manner.

800 µg of total lysate of U87MG cells transduced with either antisense-*miR-19b* (αmiR19b) or anti-scramble control (αscr) were subjected to targeted proteome analysis using the Proteome Profiler Human Phospho-Kinase Array Kit (R&D Systems, Switzerland) as previously described [19]. Phosphoprotein data were analyzed using the Phosphorylation Core Analysis tool in Ingenuity Pathway Analysis [21] (IPA, Qiagen). This data was used to predict phosphatases potentially responsible for the observed phosphorylation changes [19].

### Flow cytometry for DNA content analysis

Cells were fixed with cold methanol and incubated with DAPI (ThermoFisher) at room temperature for 10 minutes in the dark. Cell cycle distribution was assessed using a BD LSR II SORP Flow Cytometer (BD Biosciences, USA) connected to BD FACSDiva software. Data analysis was performed using FlowJo V10 (BD Biosciences).

### TCGA data analysis

MiRNA and mRNA expression data for GBM patients were obtained from The Cancer Genome Atlas (TCGA) via the Genomic Data Commons (GDC) data portal and cBioPortal. GBM cases were annotated with the established TCGA transcriptional subtypes. Expression and subtype-specific correlation analyses were performed accordingly.

### Statistical analysis

Statistical analyses were performed using GraphPad Prism v.8.2.1 (GraphPad Software) and R v4.0.3 (RStudio). Differences between two groups were assessed using the non-parametric Mann-Whitney *U* test. A *p*-value of <0.05 was considered as statistically significant. Unless otherwise stated, at least three biological, and three technical replicates were included in each experiment.

## Results

### *MiR-19b-3p* drives TMZ resistance in GBM cells

**Fig. 1:**
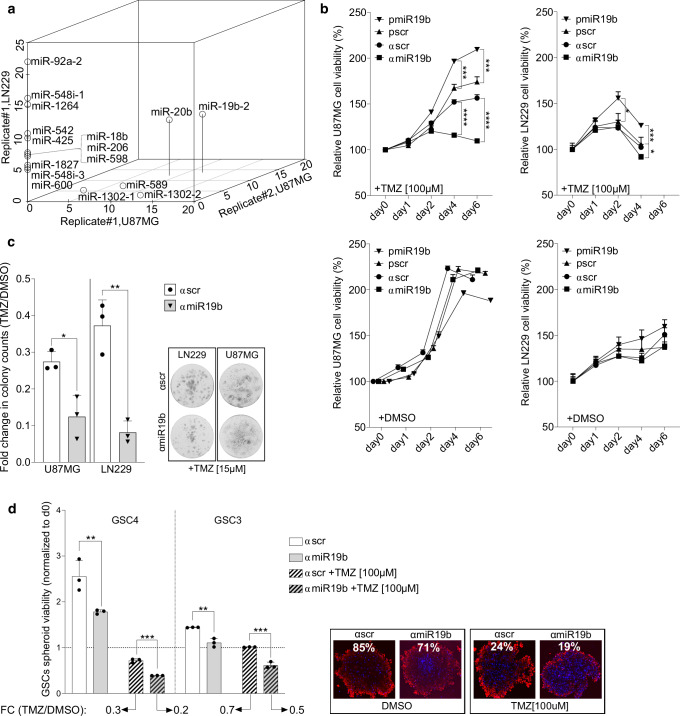
Systematic miRNA screen links *miR-19b* to TMZ resistance. **a** U87MG (x- and z-axes) and LN229 (y-axis) cells transduced with a pooled pre-miRNA library. MiRNA clones enriched >5-fold following TMZ treatment relative to the DMSO control are indicated (*p* < 0.028). **b** Cell viability (alamarBlue) of U87MG and LN229 cells transduced with *miR-19b* constructs in the presence of TMZ (upper) or DMSO control (lower). **c** Colony formation of *miR-19b*-attenuated or scrambled control cells following 15 μM TMZ treatment (*n* = 3) for 14 days, with representative images of crystal violet-stained colonies (right). **d** Cell viability GSC3 and GSC4 spheroid cultures 6 days after treatment with DMSO or TMZ [100 µM] (*n *= 3), normalised to treatment start (day0). Fold change of TMZ response relative to DMSO control is indicated at the base of the TMZ-treated bars (hatched). Right: representative overlay images of z-projected GSC4 spheroid stacks (*n* = 15). Viable outer layer cells are shown in red (calcein-AM-red-positive); dead core cells are shown in blue (Helix NP Blue-positive). Percent viable cells are indicated within the images.

To identify signaling pathways involved in TMZ resistance that are regulated by miRNAs, U87MG and LN229 GBM cell lines were transduced with a pooled lentiviral expression library comprising 520 miRNA precursors (pre-miRNAs). TMZ-resistant cells were subsequently selected through exposure to escalating doses of the drug. No viable cells were observed in the non-transduced group after TMZ treatment (supplementary Fig. 1A). Analysis of PCR products from lentiviral pre-miRNA regions revealed a discrete fragment pattern, indicating selection, 46 days post TMZ treatment (supplementary Fig. 1B). Comparison of the normalised coverage of individual pre-miRNA sequences by Next-generation sequencing revealed significant enrichment of *miR-19b* and *miR-20b*, which form part of the same pre-miRNA, in two independent U87MG screens and one LN229 screen. In contrast, other pre-miRNAs were enriched exclusively in either U87MG or LN229 cultures (Fig. 1a).

To validate the results of the lentiviral screen, individual expression constructs for pre-miRNAs identified in the screens were used to transduce U87MG cells, which were then analyzed for TMZ response. Additionally, *pre-miR-19b-2* (pmiR19b) and *pre-miR-20b* were overexpressed individually in U87MG cells. In agreement with the screen data, all identified pre-miRNAs including *miR-1302, miR-589, miR-20b, miR-19b*, and *miR-19b/20b* conferred resistance to TMZ (supplementary Fig. 1C). Notably, overexpression of *miR-19b* alone conferred improved cell viability in the presence of TMZ compared to *miR-20b* alone or *miR-19b/20b* combination (supplementary Fig. 1C). We therefore focused on *miR-19b* for subsequent experiments.

We then undertook a series of tests to substantiate the contribution of *miR-19b* to TMZ response. Overexpression of pmiR19b markedly enhanced the survival of both U87MG and LN229 cells upon treatment with TMZ, whereas overexpression of antisense *miR-19b* (αmiR19b) reduced viability compared to precursor scrambled control (pscr) or antisense scrambled control (αscr), respectively (supplementary Fig. 1D, Fig. 1b, upper panels). The relatively modest effect observed in *miR-19b*-attenuated LN229 cells was attributed to the low endogenous expression level of *miR-19* in the parental LN229 cell line (supplementary Fig. 1E). No difference in cell growth was observed in DMSO control (Fig. 1b, bottom panels). Consistent with the viability assay results, attenuation of *miR-19b* in U87MG or LN229 cells led to a marked reduction in clonogenic growth following two weeks of TMZ treatment (Fig. 1c).

Patient derived primary GBM stem cells (GSCs) are known to exhibit intrinsic resistance to radio-chemotherapy, primarily due to their enhanced DNA repair capacity and reduced susceptibility to cell death [22]. To assess therapy response in a more clinically representative context, we analyzed spheroid cultures from GSCs. Notably, spheroids from *miR-19b*-attenuated GSC3 and GSC4 cultures displayed significantly reduced viability upon temozolomide treatment (Fig. 1d). These findings support the role of *miR-19b* in mediating TMZ treatment response in GBM cells.

### *MiR-19b* confers TMZ resistance by targeting *PPP2R5E* subunit of PP2A

**Fig. 2:**
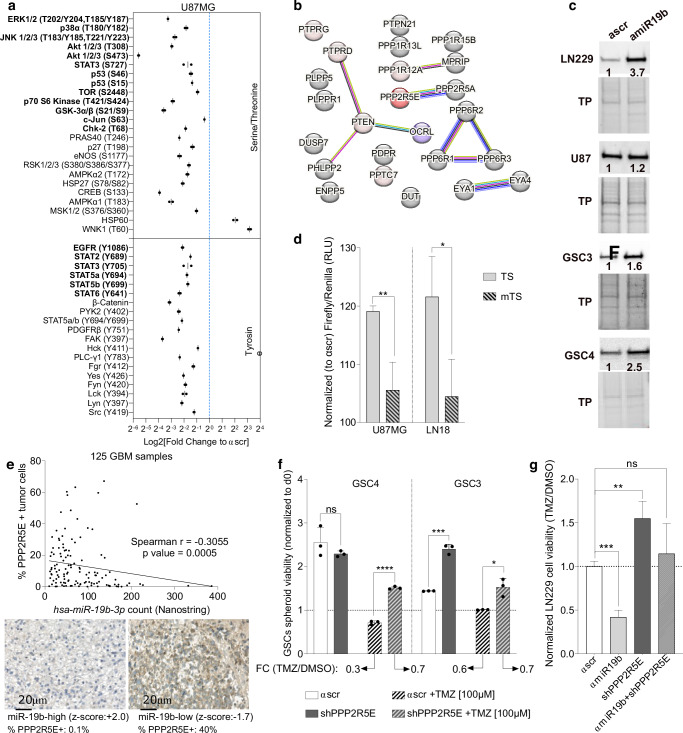
*MiR-19b* targets PP2A regulatory subunit, *PPP2R5E*. **a** Phosphoprotein array of total cell lysates from αmiR19b–transduced U87MG cells compared to αscr control cells. Log_2_ signal intensity of duplicate spots is indicated. **b** In silico-predicted phosphatase targets of *miR-19b*. Phosphatases with a target score>90 (predicted by miRDB) are shown in red; darker red indicates higher target score. Network generated using STRING. **c** Immunoblot analysis of PPP2R5E expression in *miR-19b*-attenuated cells, normalised to total protein. **d** Luciferase reporter assay in αmiR19b cells transduced with constructs containing either the wild-type 3’-UTR of *PPP2R5E* (TS), or a mutated *miR-19b* target site (mTS) (*n* = 4). Values normalised to αscr control. RLU: Relative Luminescence Unit. **e** Inverse Pearson correlation between normalised NanoString counts of *hsa-miR-19b-3p* (x-axis) and the percentage of PPP2R5E-positive cells by IHC (y-axis) (*n* = 125). Bottom: Representative images from TMA cores showing high *miR-19b* (z-score: +2) with low PPP2R5E expression (left) and low *miR-19b* (z-score -1.7) with high PPP2R5E expression. **f** Cell viability GSC4 and GSC3 spheroid cultures 6 days after treatment with DMSO or TMZ [100 µM] (*n* = 3), as performed in Fig.1D. **g** Cell viability (alamarBlue) in presence of TMZ, relative to the DMSO-treated control (*n* = 3).

To assess whether *miR-19b* modulates the activity of signaling pathways, the phosphorylation profile of kinases and effectors was analyzed in αmiR19b-tranduced U87MG cells compared to αscr using a phosphokinase antibody array. Multiple serine/threonine (S/T) and tyrosine phosphoproteins including kinases of the EGFR pathway (EGFR, ERK1/2, p38a, JNK1/2/3, AKT1/2/3 and STATs) and downstream effectors (p53, mTOR, S6 kinase, GSK-3a/b, c-Jun and Chk-2) were reduced (Fig. 2a), indicating that one or several protein phosphatases are potentially targeted by *miR-19b*. In silico predictions using miR-DB [23] suggested that among all putative phosphatase targets of *miR-19b*, *PPP2R5E*, a subunit of serine/threonine phosphatase PP2A has the highest target score (Fig. 2b). While PP2A phosphatases are known mediators of many cancer-driven kinases [24], IPA revealed that PPP2R5E-containing PP2A subunits are more specifically involved in perturbing the phosphoproteome by targeting beta catenin (supplementary Fig. 2A), a known mediator of resistance mechanisms in GBM [25]. Pathways analysis of the phosphoarray data using IPA revealed that multiple pathways involved in DNA damage, cell cycle control, senescence, and ferroptosis are affected (supplementary Fig. 2B). Attenuation of *miR-19b* in GBM cell lines and GSCs led to elevated *PPP2R5E* (Fig. 2c, supplementary Fig. 2C). Additionally, luciferase reporter assays using a construct containing the *miR-19b* target site (TS) of *PPP2R5E*, demonstrated significantly increased luciferase activity in the αmiR19b group compared to αsrc, whereas a construct with a mutated *miR-19b* target site (mTS) did not show enhanced luciferase activity (Fig. 2d). These results confirm that *PPP2R5E* is a direct target of *miR-19b* in GBM.

We next assessed the correlation between *miR-19b* and PPP2R5E expression using a tissue microarray comprising primary and recurrent glioblastoma samples (*n* = 125) [20]. PPP2R5E expression, assessed by IHC, was inversely correlated with *miR-19b* expression measured by NanoString analysis (*p* = 0.0005) (Fig. 2e). Consistently, the expression of *PPP2R5E* was inversely correlated with the expression of *miR-19b* in the highly aggressive mesenchymal molecular subtype of TCGA-GBM public dataset, but not in the less aggressive classical, proneural, or neural subtypes of GBM (supplementary Fig. 2D). Patients with low expression of *PPP2R5E* (z-score < -2) were also more frequent in the mesenchymal (57%) and neural (52%) subtypes compared to the classical (26%) and proneural (39%) subtypes, respectively (supplementary Fig. 2E). In line with the findings from *miR-19b* attenuation, in vitro silencing of PP2R5E in GSCs using two distinct shRNAs significantly increased resistance to TMZ in spheroids (supplementary Fig. 2F, Fig. 2f).

To unequivocally determine whether *miR-19b* mediates poor TMZ response through targeting *PPP2R5E*, LN229 cells were co-transduced with αmiR19b and shPPP2R5E and treated with TMZ. While αmiR19b alone increased TMZ sensitivity and shPPP2R5E alone promoted resistance, co-transduction (αmiR19b+shPPP2R5E) mitigated the individual effects of each construct (Fig. 2g), indicating that *miR-19b* is involved in TMZ response by targeting *PPP2R5E*.

We then aimed to pharmacologically activate PP2A using FTY720 (fingolimod), a small PP2A activator used in the treatment of multiple sclerosis that activates PP2A holoenzymes independent of *miR-19b* [26]. Combination treatment of GSC3 and GSC4 with TMZ and FTY720 showed more potent cytotoxic effects than individual treatments (supplementary Fig. 2G) indicating that PP2A reactivation via means other than *miR-19b* attenuation, also potentiates TMZ response in vitro.

### The *miR-19b*/PPP2R5E axis regulates temozolomide sensitivity in vivo

**Fig. 3:**
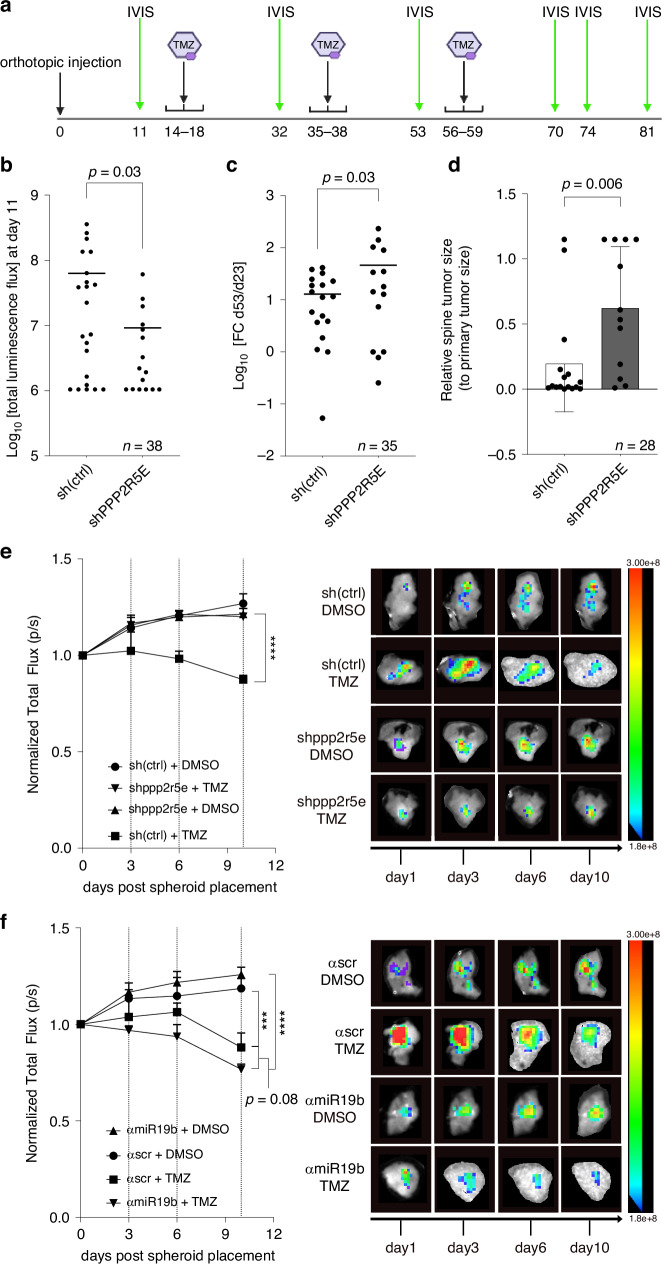
The *miR-19b*/PPP2R5E axis drives tumor progression and TMZ resistance in PDX and syngeneic glioma models. **a** Schematic of TMZ treatment cycles and timeline for in vivo tumor size monitoring (IVIS). **b** Tumor volume measured by the IVIS 11 days post-xenografting, prior to TMZ treatment. **c** Tumor volume at 53 days post-xenografting, following two TMZ treatment cycles, normalised to tumor size at day 23. **d** Tumor signal at metastatic sites relative to primary tumor signal at day 11 post-xenografting. Luminescence signal of tissue-infiltrated CT2A cells transduced with murine shpppp2r5e (**e**) or αmir19b (**f**), normalised to day0 (*n* = 3). Right: Representative image of one tissue slice per condition (+/- TMZ) and corresponding luminescence signal throughout the ex vivo experiment.

We next analyzed a patient-derived xenograft (PDX) mouse model using GSC4 cells orthotopically injected into the right brain hemisphere of nude mice to assess TMZ response in the brain tissue. To this end, sh(ctrl)-, αmiR19b-, and shPPP2R5E-GSC4 cells were transduced with beetle luciferase reporter construct allowing for monitoring tumor growth. Mice received four to five cycles of TMZ per week, beginning two weeks after tumor engraftment. This treatment regimen was repeated at 5 and 8 weeks following the protocol typically used in patients (Fig. 3a).

Unexpectedly, tumor engraftment prior to initiating therapy was compromised in mice receiving shPPP2R5E GSC4, as indicated by a smaller tumor volume 11 days post xenografting compared to the sh(ctrl) group (Fig. 3b). To account for the variability in initial tumor formation (particularly the delayed and smaller tumor onset observed in shPPP2R5E-transduced mice), tumor volumes were normalised to their respective baseline measurements at day 23 post-xenografting, when tumors were established across all groups. Tumor size was then assessed at day 53, following two cycles of TMZ treatment. Notably, the relative increase in tumor volume over this period was significantly greater in the shPPP2R5E group compared to the sh(ctrl) group (Fig. 3c), suggesting enhanced resistance to TMZ in the absence of PPP2R5E. Furthermore, shPPP2R5E tumors appeared more aggressive, as indicated by a trend towards a higher proportion of Ki-67- and β-catenin-positive tumor cells (supplementary Fig. 3A). RT-qPCR and IHC confirmed reduced PPP2R5E expression in relapsing shPPP2R5E tumors (supplementary Fig. 3B and 3C, ex vivo). Of note, GSC4 tumors formed distant metastases into the left-brain hemisphere and distant spinal cord (supplementary Fig. 3D, E). While there were no significant differences in the frequency of metastases from shPPP2R5E versus sh(ctrl) tumors, the tumor volume was larger in shPPP2R5E tumors compared to sh(ctrl) at the spinal metastatic sites (Fig. 3d), consistent with a more aggressive phenotype of *PPP2R5E*-attenuated cells.

Although *PPP2R5E* expression was significantly elevated in αmiR19b cells prior to engraftment (supplementary Fig. 3C, in vitro), it was reduced 60 days post-engraftment (supplementary Fig. 3C, ex vivo), suggesting that cells maintaining *miR-19b* knockdown were either negatively selected or reverted expression in vivo. Consistently, αmiR19b tumors were smaller than control tumors shortly after engraftment (supplementary Fig. 3F). These findings suggest that sustained *miR-19b* suppression may impair tumor growth in vivo.

To address the delayed onset of shPPP2R5E tumors and the rapid adaptive changes observed in *miR-19b*-attenuated tumors, we employed a syngeneic organotypic mouse brain model. Consistent with the results from human GSCs in vitro, attenuation of murine *mir-19b* reduced spheroid viability while *ppp2r5e* knockdown enhanced viability in CT2A murine GBM cells following TMZ treatment in vitro (supplementary Fig. 3G-H). In the next step, CT2A cells were cultured as spheroids, and placed onto brain slices from the same mouse strain, allowing them to infiltrate the tissue prior to initiating TMZ treatment (supplementary Fig. 3I-J). Importantly, tissue slices maintained high viability ( > 80%) during the course of the experiment (supplementary Fig. 3K). Luminescence imaging demonstrated that shppp2r5e spheroids were unresponsive to TMZ. In contrast, sh(ctrl) cells exhibited a marked reduction in viability upon TMZ exposure (Fig. 3e), confirming the role of *ppp2r5e* knockdown in mediating TMZ resistance. Conversely, αmir-19b CT2A spheroids were more sensitive to TMZ compared to their corresponding control (Fig. 3f). In conclusion, TMZ response is also regulated by the *mir-19b*/ppp2r5e axis in the mouse brain tissue.

### Enhanced PP2A expression induces DNA damage

**Fig. 4:**
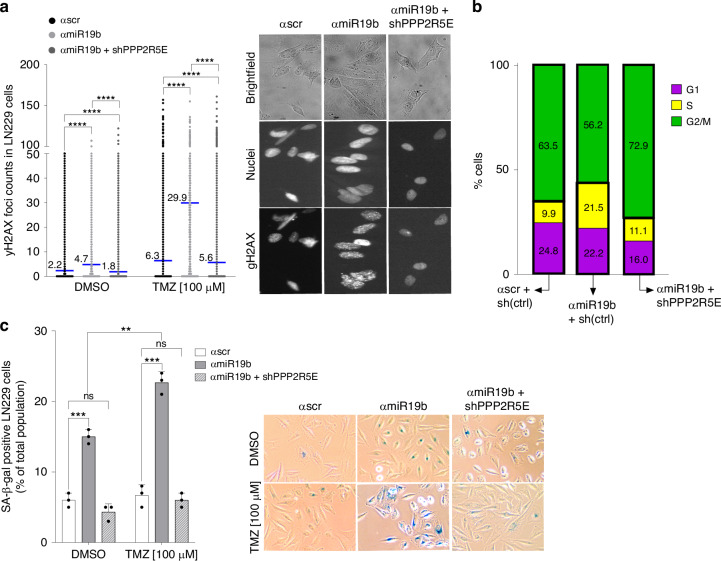
Combined *miR-19b* Inhibition and TMZ Treatment amplifies DNA Damage. **a** γH2AX foci quantification in LN229 cells treated with DMSO or TMZ for 48 h (left). Representative images at 60X magnification show bright field, nuclear staining (DAPI), and γH2AX foci (Cy3). Foci were quantified using CellProfiler; the mean (*n* > 10000 nuclei) from two biological replicates is shown as a horizontal blue line, with value indicated. **b** Cell cycle distribution of LN229 cells transduced with αmiR19b/sh(ctrl) or αmiR19b/shPPP2R5E following TMZ treatment, with percentage of cells in each cell cycle phase indicated. **c** Percentage of senescence-associated β-galactosidase-positive DMSO or TMZ treated LN229 cells (*n* = 4) (left) and representative images in bright field (right).

Next, we investigated how the *miR-19b*/PPP2R5E axis regulates TMZ response. Phosphoprotein array analysis (supplementary Fig. 2B) pointed pathways involved in DNA damage, cell cycle regulation, senescence, and ferroptosis. Focusing on DNA damage, we assessed γH2AX foci formation, a marker of TMZ-induced double-strand breaks [27]. TMZ treatment led to a striking increase in γH2AX expression and foci formation in αmiR19b LN229 and U87MG cells (Fig. 4a, supplementary Fig. 4A–B), whereas this maximised TMZ response was markedly rescued in the αmiR19b/shPPP2R5E cells (Fig. 4a).

Similar results, although at lower foci counts, were observed in DMSO-treated LN229 cells (Fig. 4a, left), U87MG cells (supplementary Fig. 4B,left), and GSC4 cells (supplementary Fig. 4C), suggesting that the *miR-19b/*PPP2R5E axis may modulate baseline genotoxic stress. Consistent with the γH2AX data, TMZ-induced phosphorylation of DNA damage checkpoint kinases CHEK1 and CHEK2 were enhanced in αmiR19b GSC cells but diminished in shPPP2R5E cells (supplementary Fig. 4D). In line with its role in activation of PP2A subunits, FTY720 treatment further amplified TMZ-induced phosphorylation of CHEK1 and CHEK2, an effect again rescued in shPPP2R5E cells (supplementary Fig. 4D). In summary, enhanced DNA damage is at least in part driven by the *miR-19b*/PPP2R5E axis.

Following extensive DNA DSB breaks, TMZ induces cell cycle arrest in G2/M [28]. TMZ treatment for two days induced G2/M arrest in approximately 50% of LN229 and U87MG cells (supplementary Fig. 4E,αscr). Modulating *miR-19b* levels altered this response, as its overexpression increased G2/M accumulation to 56–65%, while its inhibition reduced it to 31–38% (supplementary Fig. 4E). Similarly, shPPP2R5E phenocopied *miR-19b* overexpression, driving up to 60% of cells into G2/M (supplementary Fig. 4F), whereas dual targeting of *miR-19b* and its target, PPP2R5E (αmiR19b+shPPP2R5E) neutralised these opposing effects (Fig. 4b). In conclusion, although TMZ induces a canonical G2/M arrest, genotoxic stress induced by *miR-19b* inhibition shifts the arrest towards G1/S through PPP2R5E reactivation.

αmiR19b cells showed enlarged cellular and nuclear morphologies with a granular texture reminiscent of senescent cells (Fig. 4a, brightfield channel). Indeed, αmiR19b cells expressed elevated senescence-associated β-galactosidase (SA-β-gal) compared to αscr at steady-state, an effect reversed by PPP2R5E knockdown (Fig. 4c, supplementary Fig. 4G). TMZ treatment strongly induced SA-β-gal expression across all conditions, however, highest level of TMZ-induced senescence was induced in *miR-19b*-attenuated cells (Fig. 4c, supplementary Fig. 4G). These findings indicate that restoring PPP2R5E expression re-sensitises cells to genotoxic and TMZ-driven senescence.

### PPP2R5E activation contributes to ferroptosis sensitivity

**Fig. 5:**
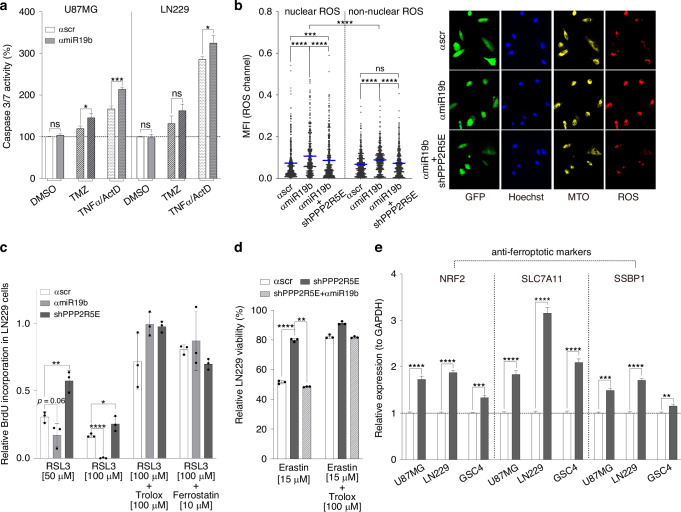
Contribution of the αmiR19b/*PPP2R5E* axis to ROS production and ferroptosis. **a** Apoptosis induced by 200 μM TMZ, or TNFα [10 ng/ml]/ ActD [0.5 ugr/ml] measured by caspase 3/7 activity assay. Values are normalised to untreated αscr control. **b** ROS production measured by confocal fluorescence microscopy. Left: Mean fluorescence intensity (MFI) for both nuclear and non-nuclear ROS. Right: Representative images of transduced cells (GFP-positive), nuclei (Hoechst), mitochondria (MitoTracker Orange, MTO), and ROS (CellRox Deep Red). **c** Cell proliferation (BrdU incorporation assay) of the LN229 αscr, αmiR19b, and shPPP2R5E cells after 48 h treatment with 50 μM or 100 μM RSL3, alone or in combination with trolox and ferrostatin. BrdU incorporation is normalised to untreated αscr control. **d** Cell viability (alamarBlue assay) of LN229 cells treated with 15 μM erastin ± 100 μM Trolox, normalized to untreated control (*n* = 4). **e** Expression of anti-ferroptotic markers in αscr and shPPP2R5E cells relative to *GAPDH*, by RTqPCR (*n* = 3).

Building on our observation that *miR-19b* attenuation induces DNA damage and senescence, we next analyzed the mechanism of cell death induced by attenuation of *miR-19b*. Surprisingly, despite extensive DNA damage accumulation, αmiR19b alone did not trigger significant apoptosis, with only minimal induction observed in a cell line-dependent manner (Fig. 5a). Notably, however, when apoptosis was extrinsically stimulated using Tumor Necrosis Factor α and Actinomycin D (TNFα/ActD), αmiR19b cells exhibited markedly increased apoptotic response (Fig. 5a).

Given the limited apoptotic response, we next investigated whether alternative cell death pathways are modulated by the *miR-19b*/PPP2R5E axis, focusing on ferroptosis, a regulated form of cell death driven by extensive lipid peroxidation and reactive oxygen species (ROS) accumulation [29, 30]. Using confocal fluorescence microscopy, we monitored intercellular ROS levels in nuclear and non-nuclear compartments (supplementary Fig. 5A). Attenuation of *miR-19b* markedly increased ROS in both compartments, particularly in the nucleus of the LN229 cells, an effect that was at least partially rescued by PPP2R5E knockdown (Fig. 5b). Of note, non-nuclear ROS predominantly co-localised with active mitochondrial, as shown by MitoTracker Orange (MTO) staining (Fig. 5b, right). These results indicate that interference with the *miR-19b*/PPP2R5E axis causes ROS accumulation, especially in the nucleus, a hallmark that may prime cells for subsequent ferroptotic death.

To test this, we pharmacologically induced ferroptosis using RSL3, an inhibitor of GPX4, or erastin, an inhibitor of cystine/glutamate antiporter SLC7A11. LN229 cells demonstrated a dose-dependent response to both ferroptosis inducers (supplementary Fig. 5B). Importantly, αmiR19b cells were more sensitive, while shPPP2R5E cells were more resistant to ferroptosis, as shown by reduced proliferation (Fig. 5c, supplementary Fig. 5C) and metabolic activity (supplementary Fig. 5D). Co-treatment with the lipid peroxide scavenger trolox or the ferroptosis inhibitor ferrostatin-1 completely rescued RSL3-induced cytotoxicity, confirming ferroptosis as the mechanism of cell death (Fig. 5c, supplementary Fig. 5C, D).

Furthermore, *miR-19b* knockdown suppressed the ferroptosis resistance conferred by PPP2R5E depletion, an effect that was again rescued by trolox (Fig. 5d), demonstrating that *miR-19b* and PPP2R5E functionally interact to control ferroptosis response.

To further validate PPP2R5E as a negative regulator of ferroptosis, we analyzed the expression of anti-ferroptotic markers *NRF2* [31, 32], *SLC7A11* [33] and *SSBP1* [34]. Indeed, all three markers were consistently upregulated in shPPP2R5E cells across multiple lines and GSCs, confirming the protective role of *miR-19b*-mediated PPP2R5E suppression against ferroptosis (Fig. 5e).

In summary, our data identified the *miR-19b*/PPP2R5E axis as a regulator of ferroptotic vulnerability, contributing to TMZ-induced cytotoxicity.

## Discussion

The mechanisms underlying poor TMZ treatment response in GBM remain poorly understood. To identify dysregulated pathways contributing to TMZ response, we conducted functional miRNA screens and identified *miR-19b* as a key regulator of TMZ treatment response. *MiR-19b* is significantly upregulated in glioma tissues and positively correlates with the glioma grade [35]. Its elevated expression is associated with poor prognosis in GBM patients [36, 37]. However, the underlying mechanisms contributing to TMZ treatment response remains so far uninvestigated.

**Fig. 6:**
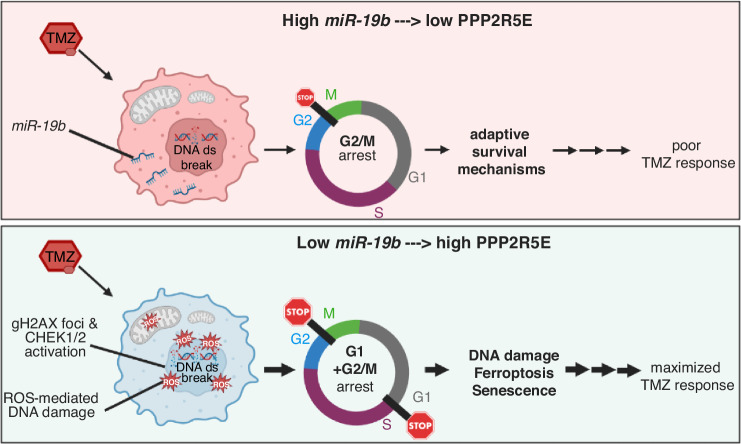
Model of the combined effect of temozolomide-induced DNA damage and PPP2R5E activation. Attenuation of *miR-19b* or treatment with FTY720 reactivates PPP2R5E-containing PP2A complexes, which synergises with TMZ by enhancing both TMZ-induced and ROS-mediated DNA damage. This cumulative stress promotes cell cycle arrest in both G1 and G2/M phases, ferroptosis, and senescence, thereby maximizing TMZ cytotoxicity.

Here we identify a previously unrecognised *miR-19b*/PPP2R5E axis that modulates TMZ response in GBM. Specifically, attenuation of *miR-19b* leads to upregulation of PPP2R5E, a regulatory subunit of the PP2A complexes, which in turn enhances DNA damage through increased production of ROS, thereby potentiating TMZ cytotoxicity (Fig. 6). While TMZ typically induces G2 cell cycle arrest [38], ROS-mediated DNA damage is known to cause arrest in the G1 [39] and S phases [40]. Consistent with this, TMZ treated *miR-19b*-attenuated cells exhibit arrest across G1, S, and G2 phases, reflecting the combined cytotoxic effects of DNA alkylation and ROS-mediated damage. Conversely, TMZ treatment of cells overexpressing *miR-19b* (pmiR-19b) or deficient in its target, PPP2R5E (shPPP2R5E) -both associated with reduced ferroptosis- results in predominant G2 phase arrest (supplementary Fig. 4E, F).

Our finding that induced expression of PPP2R5E, resulting from *miR-19b* attenuation, enhances γH2AX foci formation as well as CHEK1 and CHEK2 phosphorylation contrasts with previous reports suggesting that increased PP2A activity promotes dephosphorylation of these targets [41, 42]. However, we demonstrate that upregulation of PPP2R5E leads to excessive nuclear ROS production, resulting in DNA damage, and that these effects are further amplified upon treatment with TMZ (Fig. 6). We therefore propose that the cytotoxic stress induced by PPP2R5E expression outweighs the dephosphorylating activity of PP2A on γH2AX, CHEK1, and CHEK2. Notably, previous studies did not specifically examine PP2A complexes containing PPP2R5E, raising the possibility that other PP2A regulatory subunits are primarily responsible for dephosphorylation of these targets.

The mechanism by which PP2A activity impacts nuclear and mitochondrial ROS production is not well defined. Recently, Denisova *et al*. showed that PP2A reactivation induces inner mitochondrial membrane proton leakage, thereby increased mitochondrial ROS [43]. In hepatocellular carcinoma cells, PP2A complexes interact with GPX4, mediating its degradation in a cell-type-specific manner, providing a potential link of PP2A to enhanced ROS production and ferroptosis susceptibility [44]. PP2A activity has been linked to enhanced senescence in melanoma [45], KRAS-driven lung cancer [46] and GBM [47], however, it has not been investigated whether this is due to increased ferroptosis. Consistent with our findings, FTY720 inhibits the NRF2/ARE pathway, thereby sensitizing glioblastoma cells to TMZ response [48]. However, the mechanism of enhanced DNA damage elicited by PPP2R5E has not been addressed in previous studies. To our knowledge, we are the first to demonstrate that enhanced activity of PPP2R5E-containing PP2A complexes causes DNA damage, cellular senescence, and ferroptosis sensitivity, thereby potentiating the cytotoxic effect of TMZ.

Beyond PPP2R5E, *miR-19b* has been reported to regulate multiple targets, including PTEN, SOCS1, and BIM, thereby affecting PI3K/AKT and STAT3 signalling pathways and modulating apoptotic sensitivity [19] as well as temozolomide resistance [24]. In addition, members of the *miR-17-92* cluster, including *miR-19b*, have been implicated in metabolic rewiring and oxidative stress regulation [49], suggesting that parallel or compensatory mechanisms may contribute to the broader phenotype associated with *miR-19b* dysregulation. Thus, while our functional and mechanistic data identified PPP2R5E as a critical target of *miR-19b*, additional targets may contribute TMZ response. Future studies will be required to systematically dissect the relative contributions of these pathways.

The combined cytotoxic effects of DNA alkylation and ROS-mediated damage observed in *miR-19b*-attenuated cells, and the reversal of these effects upon PPP2R5E attenuation, was confirmed in spheroid cultures of GSC cells and syngeneic organotypic mouse model. Unexpectedly, tumor engraftment in the orthotopic PDX mouse model was impaired in shPPP2R5E GSC4 recipient mice, suggesting that establishment of the GBM niche or adaptation to the murine microenvironment may be disturbed by PPP2R5E repression. However, once established, these tumors exhibited a more aggressive phenotype. In contrast, the therapeutic response of *miR-19b*-attenuated cells could not be assessed in the PDX model due to the transient nature of the effect, suggesting that *miR-19b* may not represent a viable target for sustained therapeutic intervention.

Our results suggest that reactivating PPP2R5E containing PP2A complexes could serve as a therapeutic strategy to sensitise tumors to TMZ. Preclinical trials have demonstrated the therapeutic potential of FTY720 in GBM cell lines treated with FTY720 in combination with temozolomide. However, this pharmacological effect was evident only in 2D cultures and not in 3D cultures [50], and experiments elucidating the underlying mechanism of these unexpected findings have remained elusive. Using a genetic approach, we demonstrate that PPP2R5E attenuation contributes to TMZ response both in 2D- and 3-D cultures, as well as in syngeneic organotypic brain slices and orthotopic mouse xenografts. Whereas FTY720 enhances multiple PP2A complexes implicated in various signaling pathways that modulate multiple cellular mechanisms (Review by Kashani [24]), targeting PPP2R5E specifically affects only a subset of PP2A-induced signaling pathways, thereby potentially reducing the likelihood of adaptive cellular processes that might otherwise evade therapeutic response. Consequently, we propose that small molecule activators of PP2A (SMAPs) that specifically target PPP2R5E/PP2A complexes, could be superior strategies to enhance the cytotoxic effect of TMZ. As an alternative strategy, we propose that modulators of nuclear ROS could be a promising approach to enhance TMZ cytotoxicity. In contrast, based on our results in mouse xenografts, silencing *miR-19b* expression appears to be a less promising strategy for achieving a sustainable enhanced TMZ response in vivo.

In conclusion, our results uncover a previously unrecognised role of the miR-19b/PPP2R5E axis in the response to TMZ, highlighting new avenues for therapeutic intervention by targeting ferroptosis resistance to enhance TMZ cytotoxicity.

## Conclusions

Our study reveals that *miR-19b* promotes temozolomide resistance in glioblastoma by directly repressing PPP2R5E, leading to sustained oncogenic signaling, reduced ROS-induced DNA damage, impaired senescence, and ferroptosis resistance. Inhibiting *miR-19b* fortifies TMZ response by activating PP2A signaling. These findings are validated in vitro, in vivo, and ex vivo, highlighting the therapeutic potential of targeting the *miR-19b*/PPP2R5E axis to overcome chemoresistance in GBM.

## Supplementary information

Supplementary Figures (updated and revised)

Supplementary Figure Legends (updated and revised)

Supplementary Table 1

## Data Availability

All data supporting the findings of this study are included in the article and its supplementary materials. The raw images and datasets generated and/or analyzed during the current study are available from the corresponding authors upon request. All data supporting the findings of this study are available on request from the corresponding authors upon request.

## References

[1] Louis DN, Perry A, Wesseling P, Brat DJ, Cree IA, Figarella-Branger D, et al. The 2021 WHO classification of tumors of the central nervous system: A summary. Neuro Oncol. 2021;23:1231–51. 34185076 10.1093/neuonc/noab106 PMC8328013

[2] Kitange GJ, Mladek AC, Carlson BL, Schroeder MA, Pokorny JL, Cen L, et al. Inhibition of histone deacetylation potentiates the evolution of acquired temozolomide resistance linked to MGMT upregulation in glioblastoma xenografts. Clin Cancer Res. 2012;18:4070–9. 22675172 10.1158/1078-0432.CCR-12-0560 PMC3716364

[3] Yamashiro K, Nakao K, Ohba S, Hirose Y. Human Glioma Cells Acquire Temozolomide Resistance After Repeated Drug Exposure *Via* DNA Mismatch Repair Dysfunction. Anticancer Res. 2020;40:1315–23. 32132028 10.21873/anticanres.14073

[4] Touat M, Li YY, Boynton AN, Spurr LF, Iorgulescu JB, Bohrson CL, et al. Mechanisms and therapeutic implications of hypermutation in gliomas. Nature. 2020;580:517–23. 32322066 10.1038/s41586-020-2209-9 PMC8235024

[5] Gil Del Alcazar CR, Todorova PK, Habib AA, Mukherjee B, Burma S. Augmented HR repair mediates acquired temozolomide resistance in glioblastoma. Mol Cancer Res. 2016;14:928–40. 27358111 10.1158/1541-7786.MCR-16-0125 PMC5065752

[6] Zhang X, Li T, Yang M, Du Q, Wang R, Fu B, et al. Acquired temozolomide resistance in MGMTlow gliomas is associated with regulation of homologous recombination repair by ROCK2. Cell Death Dis. 2022;13:138. 35145081 10.1038/s41419-022-04590-6 PMC8831658

[7] Taylor KR, Barron T, Hui A, Spitzer A, Yalçin B, Ivec AE, et al. Glioma synapses recruit mechanisms of adaptive plasticity. Nature. 2023;623:366–74. 37914930 10.1038/s41586-023-06678-1 PMC10632140

[8] Karimi E, Yu MW, Maritan SM, Perus LJM, Rezanejad M, Sorin M, et al. Single-cell spatial immune landscapes of primary and metastatic brain tumours. Nature. 2023;614:555–63. 36725935 10.1038/s41586-022-05680-3 PMC9931580

[9] Knizhnik AV, Roos WP, Nikolova T, Quiros S, Tomaszowski KH, Christmann M, et al. Survival and Death Strategies in Glioma Cells: Autophagy, Senescence and Apoptosis Triggered by a Single Type of Temozolomide-Induced DNA Damage. PLoS One. 2013;8:556–65. 10.1371/journal.pone.0055665 PMC3559438 23383259

[10] Peraza-Vega RI, Valverde M, Rojas E. Interactions between miRNAs and Double-Strand Breaks DNA Repair Genes, Pursuing a Fine-Tuning of Repair. Int J Mol Sci. 2022;23:3231. 35328651 10.3390/ijms23063231 PMC8954595

[11] Stiff T, Bayraktar S, Dama P, Stebbing J, Castellano L. CRISPR screens in 3D tumourspheres identified miR-4787-3p as a transcriptional start site miRNA essential for breast tumour-initiating cell growth. Commun Biol. 2024;7:1–11. 39003349 10.1038/s42003-024-06555-1 PMC11246431

[12] Luedi MM, Singh SK, Mosley JC, Hassan ISA, Hatami M, Gumin J, et al. Dexamethasone-mediated oncogenicity in vitro and in an animal model of glioblastoma. J Neurosurg. 2018;129:1446–55. 29328002 10.3171/2017.7.JNS17668

[13] Haemmig S, Baumgartner U, Glück A, Zbinden S, Tschan MP, Kappeler A, et al. MiR-125b controls apoptosis and temozolomide resistance by targeting TNFAIP3 and NKIRAS2 in glioblastomas. Cell Death Dis. 2014;5:e1279. 24901050 10.1038/cddis.2014.245 PMC4611719

[14] Hall MP, Woodroofe CC, Wood MG, Que I, Van’t Root M, Ridwan Y, et al. Click beetle luciferase mutant and near infrared naphthyl-luciferins for improved bioluminescence imaging. Nat Commun. 2018;9:132. 29317625 10.1038/s41467-017-02542-9 PMC5760652

[15] Frenster JD, Placantonakis DG. Bioluminescent In Vivo Imaging of Orthotopic Glioblastoma Xenografts in Mice. Methods Mol Biol. 2018;1741:191–8. 29392701 10.1007/978-1-4939-7659-1_15

[16] Remy J, Linder B, Weirauch U, Konovalova J, Marschalek R, Aigner A, et al. Inhibition of PIM1 blocks the autophagic flux to sensitize glioblastoma cells to ABT-737-induced apoptosis. Biochim Biophys Acta - Mol Cell Res. 2019;1866:175–89. 30389373 10.1016/j.bbamcr.2018.10.017

[17] Humpel C. Organotypic Brain Slice Cultures. Curr Protoc Immunol. 2018;123:1–17. 10.1002/cpim.59 30311739

[18] Phour J., Vassella E. Methods in cancer research: Assessing therapy response of spheroid cultures by life cell imaging using a Cost-Effective Live-Dead Staining Protocol. Biol Methods Protoc. 2024;12:1–5. 10.1093/biomethods/bpae060 PMC11374025 39234439

[19] Baumgartner U, Berger F, Hashemi Gheinani A, Burgener SS, Monastyrskaya K, Vassella E. miR-19b enhances proliferation and apoptosis resistance via the EGFR signaling pathway by targeting PP2A and BIM in non-small cell lung cancer. Mol Cancer. 2018;17:1–15. 29455644 10.1186/s12943-018-0781-5 PMC5817797

[20] Kashani E, Schnidrig D, Gheinani AH, Ninck MS, Zens P, Maragkou T, et al. Integrated longitudinal analysis of adult grade 4 diffuse gliomas with long-term relapse interval revealed upregulation of TGF-β signaling in recurrent tumors. Neuro Oncol. 2022;16:noac265. 10.1093/neuonc/noac220 PMC10076939 36124685

[21] Krämer A, Green J, Pollard J Jr, Tugendreich S. Causal analysis approaches in ingenuity pathway analysis. Bioinformatics. 2014;30:523–30. 24336805 10.1093/bioinformatics/btt703 PMC3928520

[22] Annovazzi L, Caldera V, Mellai M, Riganti C, Battaglia L, Chirio D, et al. The DNA damage/repair cascade in glioblastoma cell lines after chemotherapeutic agent treatment. Int J Oncol. 2015;46:2299–308. 25892134 10.3892/ijo.2015.2963 PMC4441296

[23] Chen Y, Wang X. MiRDB: An online database for prediction of functional microRNA targets. Nucleic Acids Res. 2020;48:D127–31. 31504780 10.1093/nar/gkz757 PMC6943051

[24] Kashani E, Vassella E. Pleiotropy of PP2A Phosphatases in Cancer with a Focus on Glioblastoma IDH Wildtype. Cancers (Basel). 2022;14:5227. 36358647 10.3390/cancers14215227 PMC9654311

[25] Yun EJ, Kim D, Kim S, Hsieh JT, Baek ST. Targeting Wnt/β-catenin-mediated upregulation of oncogenic NLGN3 suppresses cancer stem cells in glioblastoma. Cell Death Dis. 2023;14:1–11. 37443071 10.1038/s41419-023-05967-x PMC10344874

[26] Kappos L, Radue EW, O’Connor P, Polman C, Hohlfeld R, Calabresi P, et al. A Placebo-Controlled Trial of Oral Fingolimod in Relapsing Multiple Sclerosis. N Engl J Med. 2010;362:387–401. 20089952 10.1056/NEJMoa0909494

[27] Mariotti LG, Pirovano G, Savage KI, Ghita M, Ottolenghi A, Prise KM, et al. Use of the γ-H2AX assay to investigate DNA repair dynamics following multiple radiation exposures. PLoS One. 2013;8:1–12. 10.1371/journal.pone.0079541 PMC3843657 24312182

[28] Lang F., Cornwell J. A., Kaur K., Elmogazy O., Zhang W., Zhang M., et al. Abrogation of the G2/M checkpoint as a chemosensitization approach for alkylating agents. Neuro Oncol. 2023;26:1–14. 10.1093/neuonc/noad252 PMC11145461 38134889

[29] Hu Z, Mi Y, Qian H, Guo N, Yan A, Zhang Y, et al. A Potential Mechanism of Temozolomide Resistance in Glioma–Ferroptosis. Front Oncol. 2020;10:1–9. 32656078 10.3389/fonc.2020.00897 PMC7324762

[30] Tousignant KD, Rockstroh A, Poad BLJ, Talebi A, Young RSE, Taherian Fard A, et al. Therapy-induced lipid uptake and remodeling underpin ferroptosis hypersensitivity in prostate cancer. Cancer Metab. 2020;8:11. 32577235 10.1186/s40170-020-00217-6 PMC7304214

[31] Jiang X, Yu M, Wang WK, Zhu LY, Wang X, Jin HC, et al. The regulation and function of Nrf2 signaling in ferroptosis-activated cancer therapy. Acta Pharmacol Sin. 2024;45:2229–40. 39020084 10.1038/s41401-024-01336-2 PMC11489423

[32] Dong H, Xia Y, Jin S, Xue C, Wang Y, Hu R, et al. Nrf2 attenuates ferroptosis-mediated IIR-ALI by modulating TERT and SLC7A11. Cell Death Dis. 2021;12:1–10. 34716298 10.1038/s41419-021-04307-1 PMC8556385

[33] Li X, Zhang W, Xing Z, Hu S, Zhang G, Wang T, et al. Targeting SIRT3 sensitizes glioblastoma to ferroptosis by promoting mitophagy and inhibiting SLC7A11. Cell Death Dis. 2024;15:1–14. 38395990 10.1038/s41419-024-06558-0 PMC10891132

[34] Su J, Li Y, Liu Q, Peng G, Qin C, Li Y. Identification of SSBP1 as a ferroptosis-related biomarker of glioblastoma based on a novel mitochondria-related gene risk model and in vitro experiments. J Transl Med. 2022;20:1–19. 36180956 10.1186/s12967-022-03657-4 PMC9524046

[35] Jia Z, Wang K, Zhang A, Wang G, Kang C, Han L, et al. MiR-19a and miR-19b overexpression in gliomas. Pathol Oncol Res. 2013;19:847–53. 23824915 10.1007/s12253-013-9653-x

[36] Schnabel E, Knoll M, Schwager C, Warta R, Mock A, Campos B, et al. Prognostic value of microRNA-221/2 and 17-92 families in primary glioblastoma patients treated with postoperative radiotherapy. Int J Mol Sci. 2021;22:1–18. 10.3390/ijms22062960 PMC7998975 33803955

[37] Zhi F, Shao N, Wang R, Deng D, Xue L, Wang Q, et al. Identification of 9 serum microRNAs as potential noninvasive biomarkers of human astrocytoma. Neuro Oncol. 2015;17:383–91. 25140035 10.1093/neuonc/nou169 PMC4483096

[38] McCord M, Jamshidi P. Targeting the cell cycle to enhance chemotherapy efficacy in glioblastoma. Neuro Oncol. 2024;26:1097–8. 38517031 10.1093/neuonc/noae062 PMC11145455

[39] Sun Y, Deng R, Zhang C. Erastin induces apoptotic and ferroptotic cell death by inducing ROS accumulation by causing mitochondrial dysfunction in gastric cancer cell HGC.27. Mol Med Rep. 2020;22:2826–32. 32945484 10.3892/mmr.2020.11376 PMC7453531

[40] Zhou XY, Zhang J, Li Y, Chen YX, Wu XM, Li X, et al. Advanced Oxidation Protein Products Induce G1/G0-Phase Arrest in Ovarian Granulosa Cells via the ROS-JNK/p38 MAPK-p21 Pathway in Premature Ovarian Insufficiency. Oxid Med Cell Longev. 2021;2021:6634718. 34367464 10.1155/2021/6634718 PMC8337115

[41] Freeman AK, Dapic V, Monteiro AN. Negative regulation of CHK2 activity by protein phosphatase 2A is modulated by DNA damage. Cell Cycle. 2010;9:736–47. 20160490 10.4161/cc.9.4.10613 PMC3040716

[42] Leung-Pineda V, Ryan CE, Piwnica-Worms H. Phosphorylation of Chk1 by ATR Is Antagonized by a Chk1-Regulated Protein Phosphatase 2A Circuit. Mol Cell Biol. 2006;26:7529–38. 17015476 10.1128/MCB.00447-06 PMC1636880

[43] Denisova OV, Merisaari J, Huhtaniemi R, Qiao X, Yetukuri L, Jumppanen M, et al. PP2A-based triple-strike therapy overcomes mitochondrial apoptosis resistance in brain cancer cells. Mol Oncol. 2023;17:1803–20. 37458534 10.1002/1878-0261.13488 PMC10483611

[44] Qian B, Che L, Du ZB, Guo NJ, Wu XM, Yang L, et al. Protein phosphatase 2A-B55β mediated mitochondrial p-GPX4 dephosphorylation promoted sorafenib-induced ferroptosis in hepatocellular carcinoma via regulating p53 retrograde signaling. Theranostics. 2023;13:4288–302. 37554285 10.7150/thno.82132 PMC10405852

[45] Mannava S, Omilian AR, Wawrzyniak JA, Fink EE, Zhuang D, Miecznikowski JC, et al. PP2A-B56α controls oncogene-induced senescence in normal and tumor human melanocytic cells. Oncogene. 2012;31:1484–92. 21822300 10.1038/onc.2011.339 PMC3213274

[46] Kauko O, O’Connor CM, Kulesskiy E, Sangodkar J, Aakula A, Izadmehr S, et al. PP2A inhibition is a druggable MEK inhibitor resistance mechanism in KRAS-mutant lung cancer cells. Sci Transl Med. 2018;10:eaaq1093. 30021885 10.1126/scitranslmed.aaq1093 PMC8335581

[47] Cucinotta L, Filippone A, Casili G, Lanza M, Bova V, Capra AP, et al. The Pivotal Role of Protein Phosphatase 2A (PP2A) in Brain Tumors. Int J Mol Sci. 2022;23:15717. 36555359 10.3390/ijms232415717 PMC9779694

[48] Zhang L, Wang H. FTY720 inhibits the Nrf2/ARE pathway in human glioblastoma cell lines and sensitizes glioblastoma cells to temozolomide. Pharmacol Rep. 2017;69:1186–93. 29128799 10.1016/j.pharep.2017.07.003

[49] Izreig S, Samborska B, Johnson RM, Sergushichev A, Ma EH, Lussier C, et al. The miR-17 92 microRNA Cluster Is a Global Regulator of Tumor Metabolism Article The miR-17 92 microRNA Cluster Is a Global Regulator of Tumor Metabolism. CellReports. 2016;16:1915–28. 10.1016/j.celrep.2016.07.036 27498867

[50] Davy M, Genest L, Legrand C, Pelouin O, Froget G, Castagné V, et al. Evaluation of Temozolomide and Fingolimod Treatments in Glioblastoma Preclinical Models. Cancers (Basel). 2023;15:4478. 37760448 10.3390/cancers15184478 PMC10527257

