# Supplementary material for: Targeting the miR-19b/PPP2R5E axis enhances temozolomide response in glioblastoma via ROS-induced DNA damage
Source: Br J Cancer. 2026 May 27;135(5):685–96. doi: 10.1038/s41416-026-03474-2 (PMC13478537; doi:10.1038/s41416-026-03474-2)
Supplement: Supplementary file 1 [file 41416_2026_3474_MOESM1_ESM.pdf]

Suppl. Fig. 1

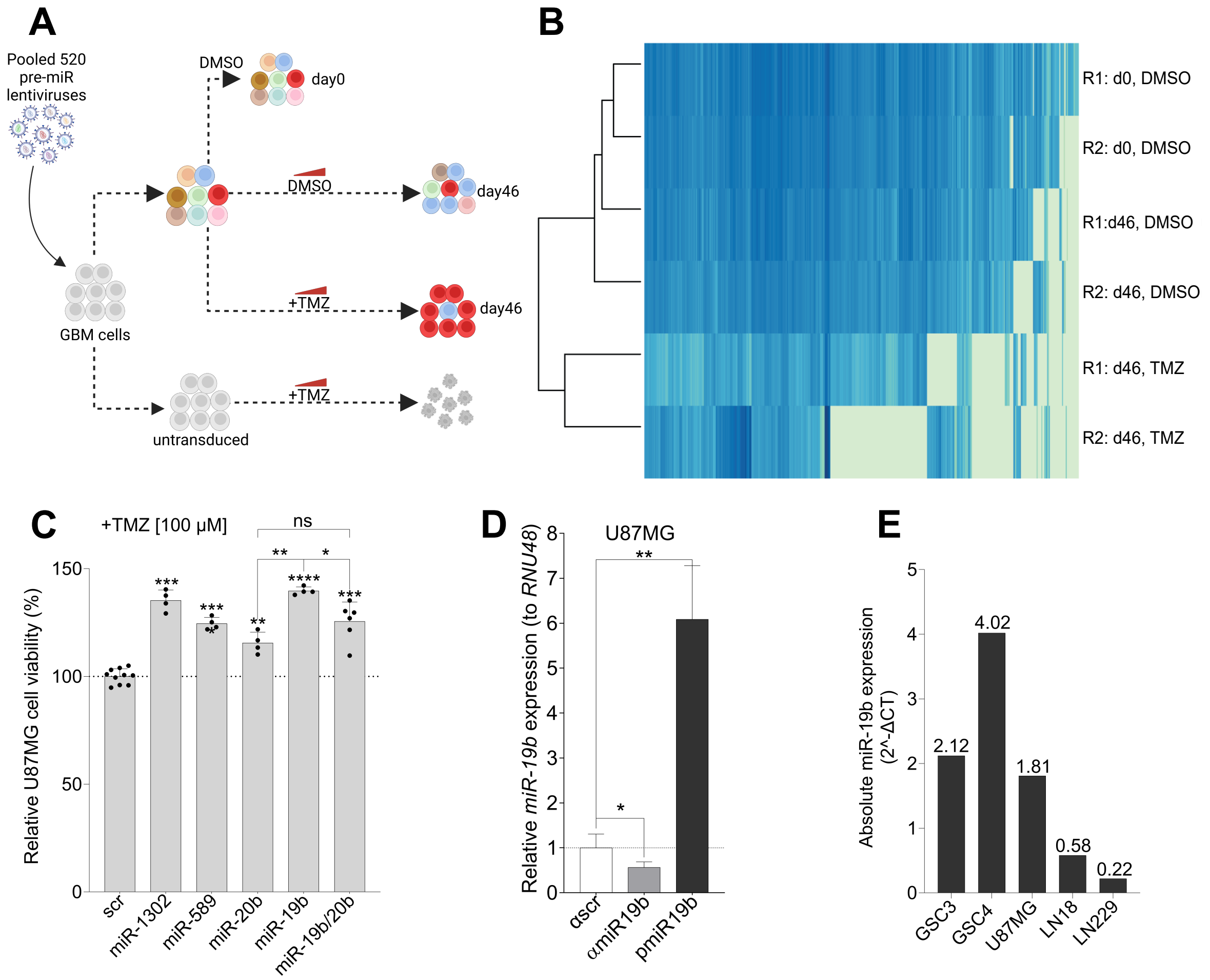
A
Pooled 520 pre-miR lentiviruses
GBM cells
DMSO
day0
DMSO
day46
+TMZ
day46
untransduced
+TMZ
B
R1: d0, DMSO
R2: d0, DMSO
R1:d46, DMSO
R2: d46, DMSO
R1: d46, TMZ
R2: d46, TMZ
C
+TMZ [100 μM]
ns
Relative U87MG cell viability (%)
150
100
50
0
scr
miR-1302
miR-589
miR-20b
miR-19b
miR-19b/20b
D
U87MG
Relative miR-19b expression (to RNU48)
αscr
αmiR19b
pmiR19b
E
Absolute miR-19b expression (2^-ΔCT)
2.12
4.02
1.81
0.58
0.22
GSC3
GSC4
U87MG
LN18
LN229

Suppl. Fig. 2

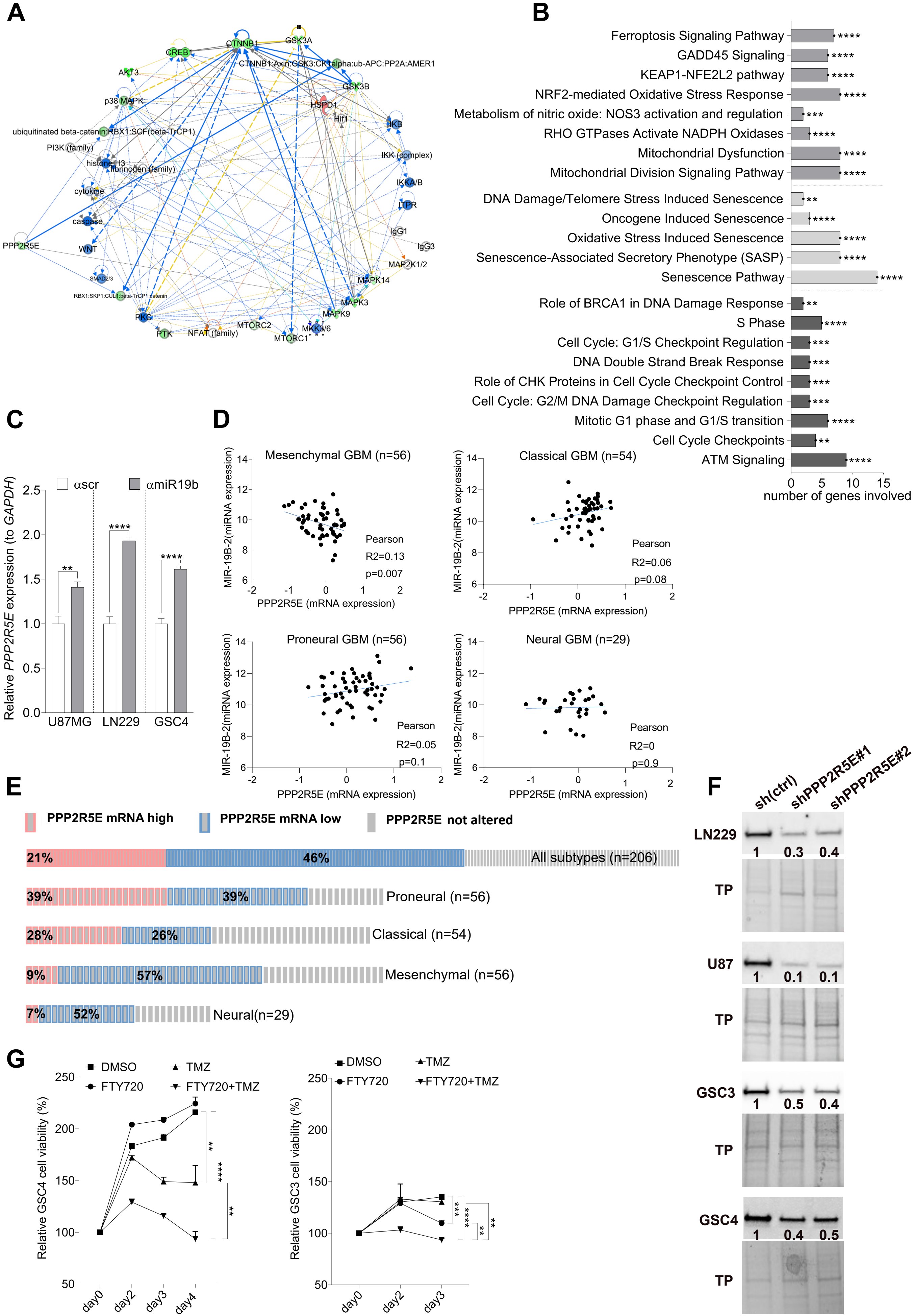
A
B
CTNNB1
GSK3A
CREB1
CTNNB1:Axin:GSK3:CK1alpha:ub-APC:PP2A:AMER1
AKT3
GSK3B
p38 MAPK
HSPD1
Hif1
ubiquitinated beta-catenin:RBX1:SCF(beta-TrCP1)
IKB
PI3K (family)
IKK (complex)
histone H3
fibrinogen (family)
IKKA/B
cytokine
ITPR
caspase
IgG1
PPP2R5E
WNT
IgG3
MAP2K1/2
SMAD2/3
MAPK14
RBX1:SKP1:CUL1:beta-TrCP1:catenin
MAPK3
PKG
MAPK9
MTORC2
MKK3/6
PTK
NFAT (family)
MTORC1
Ferroptosis Signaling Pathway ****
GADD45 Signaling ****
KEAP1-NFE2L2 pathway ****
NRF2-mediated Oxidative Stress Response ****
Metabolism of nitric oxide: NOS3 activation and regulation ***
RHO GTPases Activate NADPH Oxidases ****
Mitochondrial Dysfunction ****
Mitochondrial Division Signaling Pathway ****
DNA Damage/Telomere Stress Induced Senescence **
Oncogene Induced Senescence ****
Oxidative Stress Induced Senescence ****
Senescence-Associated Secretory Phenotype (SASP) ****
Senescence Pathway ****
Role of BRCA1 in DNA Damage Response **
S Phase ****
Cell Cycle: G1/S Checkpoint Regulation ***
DNA Double Strand Break Response ***
Role of CHK Proteins in Cell Cycle Checkpoint Control ***
Cell Cycle: G2/M DNA Damage Checkpoint Regulation ***
Mitotic G1 phase and G1/S transition ****
Cell Cycle Checkpoints **
ATM Signaling ****
0 5 10 15
number of genes involved
C
αscr
αmiR19b
Relative PPP2R5E expression (to GAPDH)
2.5
2.0
1.5
1.0
0.5
0.0
**
****
****
U87MG
LN229
GSC4
D
Mesenchymal GBM (n=56)
Pearson
R2=0.13
p=0.007
Classical GBM (n=54)
Pearson
R2=0.06
p=0.08
Proneural GBM (n=56)
Pearson
R2=0.05
p=0.1
Neural GBM (n=29)
Pearson
R2=0
p=0.9
MIR-19B-2(miRNA expression)
PPP2R5E (mRNA expression)
14
12
10
8
6
-2
-1
0
1
2
E
PPP2R5E mRNA high
PPP2R5E mRNA low
PPP2R5E not altered
21%
46%
All subtypes (n=206)
39%
39%
Proneural (n=56)
28%
26%
Classical (n=54)
9%
57%
Mesenchymal (n=56)
7%
52%
Neural(n=29)
F
sh(ctrl)
shPPP2R5E#1
shPPP2R5E#2
LN229
1 0.3 0.4
TP
U87
1 0.1 0.1
TP
GSC3
1 0.5 0.4
TP
GSC4
1 0.4 0.5
TP
G
DMSO
TMZ
FTY720
FTY720+TMZ
Relative GSC4 cell viability (%)
250
200
150
100
50
**
****
**
day0
day2
day3
day4
Relative GSC3 cell viability (%)
***
****
**
**
day0
day2
day3

Suppl. Fig. 3

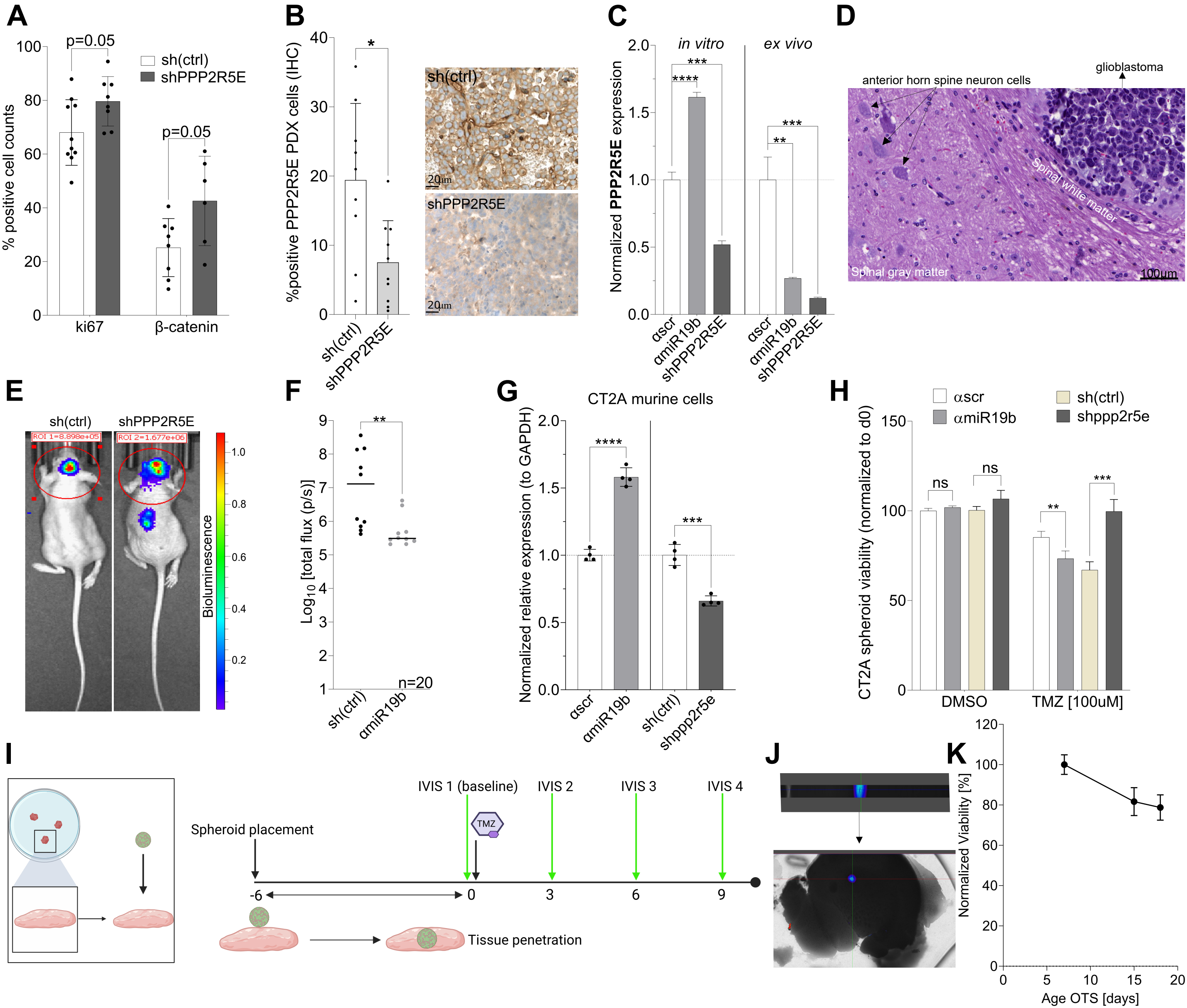

A
p=0.05
p=0.05
sh(ctrl)
shPPP2R5E
% positive cell counts
ki67
β-catenin
B
*
%positive PPP2R5E PDX cells (IHC)
sh(ctrl)
shPPP2R5E
20μm
C
in vitro
ex vivo
Normalized PPP2R5E expression
αscr
αmiR19b
shPPP2R5E
D
anterior horn spine neuron cells
glioblastoma
Spinal white matter
Spinal gray matter
100μm
E
sh(ctrl)
shPPP2R5E
ROI 1=8.898e+05
ROI 2=1.677e+06
Bioluminescence
F
**
Log10 [total flux (p/s)]
n=20
sh(ctrl)
αmiR19b
G
CT2A murine cells
Normalized relative expression (to GAPDH)
****
***
αscr
αmiR19b
sh(ctrl)
shppp2r5e
H
αscr
αmiR19b
sh(ctrl)
shppp2r5e
CT2A spheroid viability (normalized to d0)
ns
ns
**
***
DMSO
TMZ [100uM]
I
Spheroid placement
IVIS 1 (baseline)
IVIS 2
IVIS 3
IVIS 4
TMZ
-6
0
3
6
9
Tissue penetration
J
K
Normalized Viability [%]
Age OTS [days]

Suppl. Fig. 4

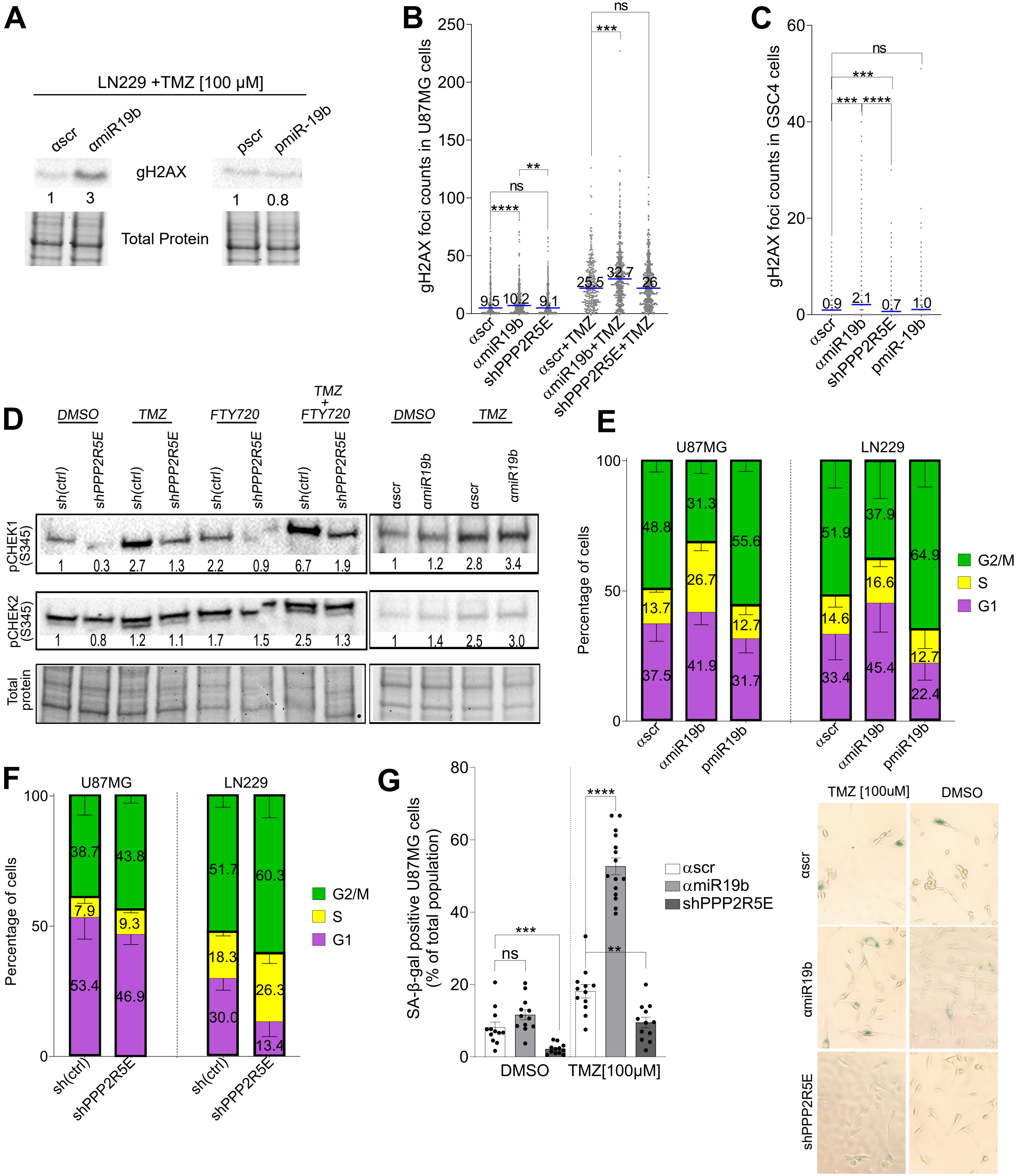
A
LN229 +TMZ [100 µM]
αscr
αmiR19b
pscr
pmiR-19b
gH2AX
1
3
1
0.8
Total Protein
B
gH2AX foci counts in U87MG cells
9.5
10.2
9.1
25.5
32.7
26
ns
ns
****
**
***
****
αscr
αmiR19b
shPPP2R5E
αscr+TMZ
αmiR19b+TMZ
shPPP2R5E+TMZ
C
gH2AX foci counts in GSC4 cells
0.9
2.1
0.7
1.0
ns
***
*** ****
αscr
αmiR19b
shPPP2R5E
pmiR-19b
D
DMSO
TMZ
FTY720
TMZ + FTY720
sh(ctrl)
shPPP2R5E
αscr
αmiR19b
pCHEK1 (S345)
1 0.3 2.7 1.3 2.2 0.9 6.7 1.9
1 1.2 2.8 3.4
pCHEK2 (S345)
1 0.8 1.2 1.1 1.7 1.5 2.5 1.3
1 1.4 2.5 3.0
Total protein
E
U87MG
LN229
Percentage of cells
48.8
31.3
55.6
13.7
26.7
12.7
37.5
41.9
31.7
51.9
37.9
64.9
14.6
16.6
12.7
33.4
45.4
22.4
G2/M
S
G1
αscr
αmiR19b
pmiR19b
F
U87MG
LN229
Percentage of cells
38.7
43.8
7.9
9.3
53.4
46.9
51.7
60.3
18.3
26.3
30.0
13.4
G2/M
S
G1
sh(ctrl)
shPPP2R5E
G
SA-β-gal positive U87MG cells (% of total population)
****
***
ns
**
αscr
αmiR19b
shPPP2R5E
DMSO
TMZ[100µM]
TMZ [100uM]
DMSO
αscr
αmiR19b
shPPP2R5E

Suppl. Fig. 5

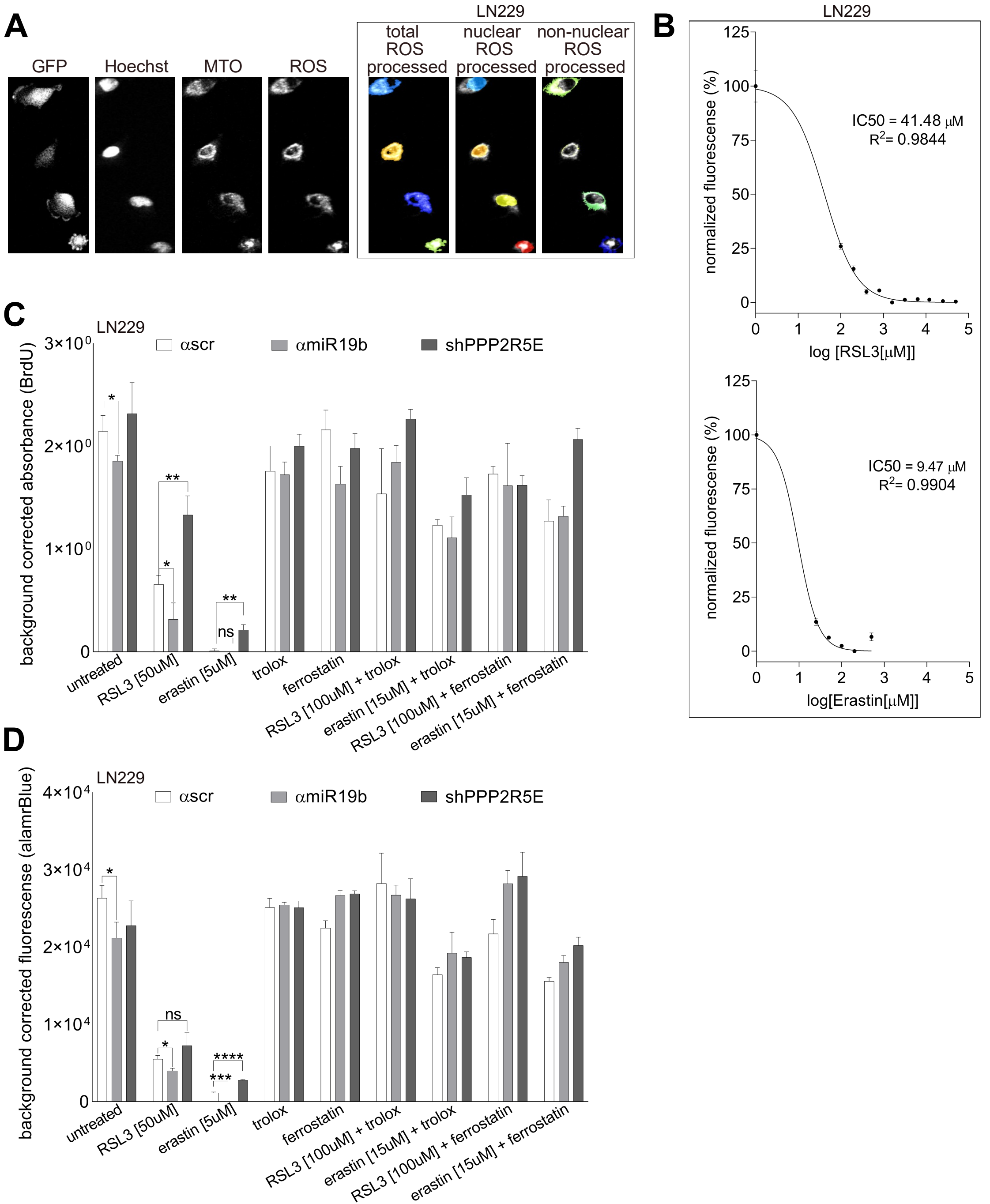

A
LN229
GFP
Hoechst
MTO
ROS
total ROS processed
nuclear ROS processed
non-nuclear ROS processed
B
LN229
normalized fluorescense (%)
IC50 = 41.48 μM
R2= 0.9844
log [RSL3[μM]]
IC50 = 9.47 μM
R2= 0.9904
log[Erastin[μM]]
C
LN229
αscr
αmiR19b
shPPP2R5E
background corrected absorbance (BrdU)
3×10^0
2×10^0
1×10^0
ns
untreated
RSL3 [50uM]
erastin [5uM]
trolox
ferrostatin
RSL3 [100uM] + trolox
erastin [15uM] + trolox
RSL3 [100uM] + ferrostatin
erastin [15uM] + ferrostatin
D
LN229
αscr
αmiR19b
shPPP2R5E
background corrected fluorescense (alamrBlue)
4×10^4
3×10^4
2×10^4
1×10^4
ns
untreated
RSL3 [50uM]
erastin [5uM]
trolox
ferrostatin
RSL3 [100uM] + trolox
erastin [15uM] + trolox
RSL3 [100uM] + ferrostatin
erastin [15uM] + ferrostatin
