# Supplementary material for: Targeting the miR-19b/PPP2R5E axis enhances temozolomide response in glioblastoma via ROS-induced DNA damage
Source: Br J Cancer. 2026 May 27;135(5):685–96. doi: 10.1038/s41416-026-03474-2 (PMC13478537; doi:10.1038/s41416-026-03474-2)
Supplement: Supplementary file 2 [file 41416_2026_3474_MOESM2_ESM.docx]

**S.Fig.1 Pooled lentiviral miRNA screen. A** Schematic of the experimental workflow used to identify miRNAs conferring TMZ resistance. Parental untransduced GBM cells are indicated in grey; cells transduced with different lentiviral constructs are indicated in various colours. Irregularly shaped cells represent dying cells. **B** Heat map of NGS results from pre-miRNA libraries shortly after transduction (day 0, DMSO), after 46 days with DMSO treatment (day 46, DMSO), and after 46 days with TMZ treatment (day 46, TMZ), in two independent U87MG replicates (R1 and R2). Each blue bar represents one specific miRNA. **C** Cell viability measured by alamarBlue assay in U87MG cells transduced with candidate pre-miRs identified in the lentiviral screen, following 72h TMZ treatment, relative to pscr control. **D** *MiR-19b* expression in U87MG cells transduced with pre-miR constructs, measured by RT-qPCR (TaqMan) and normalized to *RNU48* (n=4). **E** Endogenous levels of *miR-19b* in glioblastoma stem cells (GSCs) and GBM cell lines, measured by TaqMan RT-qPCR (n=2).

**S.Fig.2	*MiR-19b*/PPP2R5E axis links kinase signaling to TMZ response across GBM subtypes.** **A** Gene network analysis of PPP2R5E, which serves as a central hub for phosphokinases (indicated at the circle periphery). Inhibitory interactions are shown in blue; activating interactions are shown in orange, with p<0.05. **B** Pathway analysis of phosphokinase array data using the IPA tool. Significance is indicated by asterisks. Pathways are grouped by cellular process: ferroptosis-related (medium grey), senescence-related (light grey), and DNA damage/cell cycle-related (dark grey). Total number of genes: 53. **C** *PPP2R5E* expression relative to *GAPDH* in αmiR19b cells measured by RTqPCR (n=4). **D** Correlation between *miR-19b* and *PPP2R5E* mRNA levels of mesenchymal (n=56),classical (n=54), proneural (n=56), or neural (n=29) subtypes from the TCGA-GBM dataset. Pearson correlation coefficient (R^2^) and p-values are indicated. **E** Oncoprint of *PPP2R5E* expression levels in GBM subtypes (TCGA-GBM, n=206), based on z-scores. High expression: z-score > 2; low expression: z-score < -2; unaltered: -2 ≤ z ≤ 2. Percentage of high and low expression cases is indicated. **F** Western blot showing PPP2R5E expression in LN229 cells transduced with two independent shRNAs. Expression is normalized to total protein. **G** Cell viability of spheroid cultures from GSC4 and GSC3 cells transduced treated with FTY720 [10µM], TMZ [100µM], or combination for indicated days (n=3), normalized to treatment start (day0).

**S.Fig.3 *In vivo* and *ex vivo* validation of *miR-19b*/PPP2R5E impact on TMZ response A** Immunohistochemical analysis of tumors from mice euthanized at the onset of neurological symptoms. **B** Percentage of PPP2R5E-positive tumor cells in sh(ctrl) (n=9) and shPPP2R5E (n=10) PDX tumors assessed by immunohistochemistry. Right: representative images of the indicated tumors. **C** *PPP2R5E* mRNA expression in GSC4-transduced cells prior to tumor engraftment and in tumors isolated from mice following TMZ treatment for 63 days (shPPP2R5E) or 42 days (αmiR19b), quantified by SYBR Green RTqPCR and normalized to *GAPDH* (n=3). **D** H&E-stained section of a spinal cord metastasis from a shPPP2R5E tumor harvested 74 days post-xenografting. **E** Representative IVIS images of mice engrafted with sh(ctrl) and shPPP2R5E GSC4 cells, acquired 74 days post-xenografting, showing signal corresponding to spinal cord metastasis. **F** Tumor volume of αmiR19b-engrafted mice measured by IVIS imaging 11 days post-xenografting, prior to initiating TMZ treatment. **G** *Ppp2r5e* expression relative to *gapdh* in αmiR19b- and shppp2r5e-transduced CT2A murine cells measured by SYBR Green RT-qPCR (n=4). **H** Spheroid viability of ppp2r5e- or αmiR19b-attenuated CT2A cells treated with DMSO or 100µM TMZ for 6 days, normalized to day 0 (n=3). **I** Syngeneic *ex vivo* mouse model using CT2A murine glioma cells and longitudinal tumor monitoring. **J** Confocal 3D image analysis of organotypic brain tissue slices indicating spheroid penetration within the tissue slice. **K** Tissue viability was assessed using either Calcein Red-AM [1.2µM] and Helix NP Blue [2.5 µM], or a combination of Calcein-AM [2 µM] and propidium iodide [PI; 5 µg/mL], serving as independent (but parallel) viability controls.

**S.Fig.4 DNA damage is induced by inhibiting the *miR19b/PPP2R5E* axis. A** γH2AX protein expression by Western blot in LN229 ɑmiR19b or pmiR19b cells, treated with TMZ [100uM] for 2 days. Expression levels are shown relative to total protein. **B** γH2AX foci formation in αmiR19b-, shPPP2R5E-, or αscr-transduced U87MG cells following treatment with 100 µM TMZ or DMSO for 2 days. **C** γH2AX foci formation in αmiR19b, shPPP2R5E, and pmiR19b GSC4 cells in the absence of TMZ. **D** Western blot analysis of checkpoint proteins in GSC4 cells transduced with shPPP2R5E or ɑmiR19b following treatment with TMZ, FTY720, or combinations for 2 days. Indicated expression values are shown relative to total protein and normalized to the sh(ctrl) values. **E,F** Cell cycle distribution of αmiR19b or pmiR19b cells (**E**) and shPPP2R5E cells (**F**) cells following TMZ treatment (mean±SD, n=3). The percentage of cells in each phase is indicated. **G** Percentage of senescence-associated β-galactosidase-positive U87MG cells treated with DMSO or TMZ (left panel, n=4) and representative brightfield images (right panel).

**S.Fig.5 The *miR19b*/PPP2R5E axis modulates ferroptosis cell death in LN229 cells. A** Representative images from CellProfiler pipeline used for segmentation of nuclear and non-nuclear ROS signals. Left: GFP, Hoechst, MitoTracker Orange (MTO), and ROS channels of a random representative field-of-view (raw images). Right (boxed): ROS channel following compartment-specific processing of the same field-of-view. **B** IC_50_ curves of LN229 cells treated with RSL3 (top) or erastin (bottom). Viability was measured by alamarBlue assay 48h post-treatment and normalized to untreated controls. **C,D** Cell proliferation measured by BrdU incorporation assay (**C**), and cell viability measured by alamarBlue assay (**D**), in LN229 cells treated for 48h with RSL3, erastin, trolox, or ferrostatin-1, alone or in combination. Background-corrected values are shown (n=3). Trolox and ferrostatin-1 were used at final concentrations of 100µM and 10µM, respectively.
